# Analysis of factors influencing anxiety and depression disorders among hospitalized patients

**DOI:** 10.3389/fpsyt.2026.1757945

**Published:** 2026-02-27

**Authors:** Nan Wang, Qiuyun Tu

**Affiliations:** Department of Gerontology and Geriatrics, The Fifth Affiliated Hospital of Sun Yat-sen University, Zhuhai, Guangdong, China

**Keywords:** anxiety, cognitive function, depression, frailty, gender, sleep quality

## Abstract

**Background:**

As globally prevalent mental health conditions, anxiety and depression impose significant socioeconomic burdens on healthcare systems. Key risk factors for anxiety and depression were determined in the current study by examining the associations across multiple domains.

**Methods:**

Participants were enrolled for this cross-sectional study from the Department of Gerontology at the Fifth Affiliated Hospital of Sun Yat-sen University in 2023. Anxiety and depression were evaluated based on the Hamilton Anxiety (HAM-A) and Hamilton Depression (HAMD) Rating Scales, respectively. Cognitive function, sleep quality, frailty, and nutritional status were evaluated using the Montreal Cognitive Assessment (MoCA), the Pittsburgh Sleep Quality Index (PSQI), the FRAIL scale, and the Nutrition Risk Screening (NRS)-2002, respectively. Multiple linear regression analysis was performed to determine the factors associated with anxiety and depression.

**Results:**

A total of 1121 participants (659 women and 462 men; mean age ± SD: 64.67 ± 10.86 years) were studied. HAMD scores were significantly associated with gender (β = 0.078, *P* = 0.017), body mass index (β = -0.096, *P* = 0.003), MoCA (β = -0.141, *P* < 0.001), FRAIL score (β = 0.200, *P* < 0.001), PSQI (β = 0.494, *P* < 0.001), and NRS-2002 (β = 0.099, *P* = 0.004) after full adjustment. HAM-A scores exhibited significant and independent associations with gender (β = 0.155, *P* < 0.001), illness (β = -0.098, *P* = 0.006), MoCA (β = -0.114, *P* = 0.001), PSQI (β = 0.435, *P* < 0.001), and FRAIL score (β = 0.276, *P* < 0.001).

**Conclusions:**

Male patients with superior physical and cognitive function, better sleep, favorable body mass index and nutrition status, and fewer illnesses had better mood. Targeted interventions, including cognitive preservation, sleep optimization, frailty prevention, comorbidity management, and lifestyle modification, may be correlated with reduced symptom burden. Special consideration is warranted for elderly and female populations.

## 1 Introduction

Anxiety and depression are common mental health disorders worldwide. Prior to 2020, the estimated global prevalence of anxiety and depression disorders was 3.6% and 4.4%, respectively (1). These conditions impose a substantial economic and social burden on public health systems (2). Anxiety and depression impose a substantial burden among hospitalized patients, contributing to increased healthcare utilization, longer hospital stays, and poorer recovery outcomes. However, greater than one-half of these patients do not receive appropriate mental health treatment (3). Without appropriate intervention, these disorders can progress into an illness, severely impairing mental and physical health. This finding underscores the critical importance of identifying modifiable risk factors and implementing preventive interventions for anxiety and depression in the hospital setting.

The etiology of anxiety and depression involves a complex interplay of biological, environmental, temperamental, and psychological factors (4). Well-established risk factors include female gender, physical illness, cognitive impairment, limited social contact, and a history of psychiatric disorders (5–8). Previous studies have identified strong associations between mental disorders and cognitive function, frailty, and sleep quality (9–11). The prevalence of mental disorders affects sleep and increases the risk of functional and cognitive impairment, while cognitive decline, sleep disorders, and frailty exacerbate mental disorders (12–15). Given these established associations, future inferential research should rigorously control for confounding factors. The mechanisms underlying mental disorders include hypercortisolemia, chronic inflammation, and common shared risk factors, such as vascular disease, chronic illness, unhealthy lifestyles, and malnutrition (1, 16, 17). Vascular risk factors, including body mass index (BMI), lipid and blood glucose levels, blood pressure, and cigarette smoking, induce neurodegenerative changes and mood-related symptoms (18–21). The occurrence and development of anxiety and depression are driven by interactions among multiple factors. Isolated analysis of individual determinants may therefore have limited explanatory power. However, most studies have examined risk factors from a single perspective, leaving the combined effects across multiple domains poorly understood. Consequently, adopting analytical approaches that capture this multidomain evaluation represents a critical advancement for the field.

Anxiety and depression are highly prevalent among hospitalized adults, yet they often remain underrecognized and inadequately managed in routine clinical practice. Although early prevention and multidomain interventions have shown promise in alleviating or even reversing mental health disorders, the contributing factors among general hospital inpatients have not been thoroughly investigated. Existing evidence lacks a comprehensive understanding of overall risk across multiple domains in this specific setting. This study was designed to determine the correlation between depression and anxiety with multiple risk factors, including demographic, epidemiologic, lifestyle, and clinical and biological domains in hospitalized patients. Our objective was to identify the most critical risk factors for anxiety and depression to inform the design of targeted strategies for promoting mental health.

## 2 Methods

### 2.1 Study design and participants

Participants in this cross-sectional study were consecutively recruited from the Department of Gerontology at the Fifth Affiliated Hospital of Sun Yat-sen University (Zhuhai, China) from August 2023 to April 2024. Prior to recruitment, a sample size estimation was performed for the planned multiple linear regression analysis. Based on the 10–15 observations per predictor variable rule of thumb of and anticipating approximately 20 key predictors in the final model, a minimum of 200–300 participants was required. To ensure robust parameter estimation, account for missing data, and allow for model exploration, a recruitment target of 700 participants was set.

**Figure 1:**
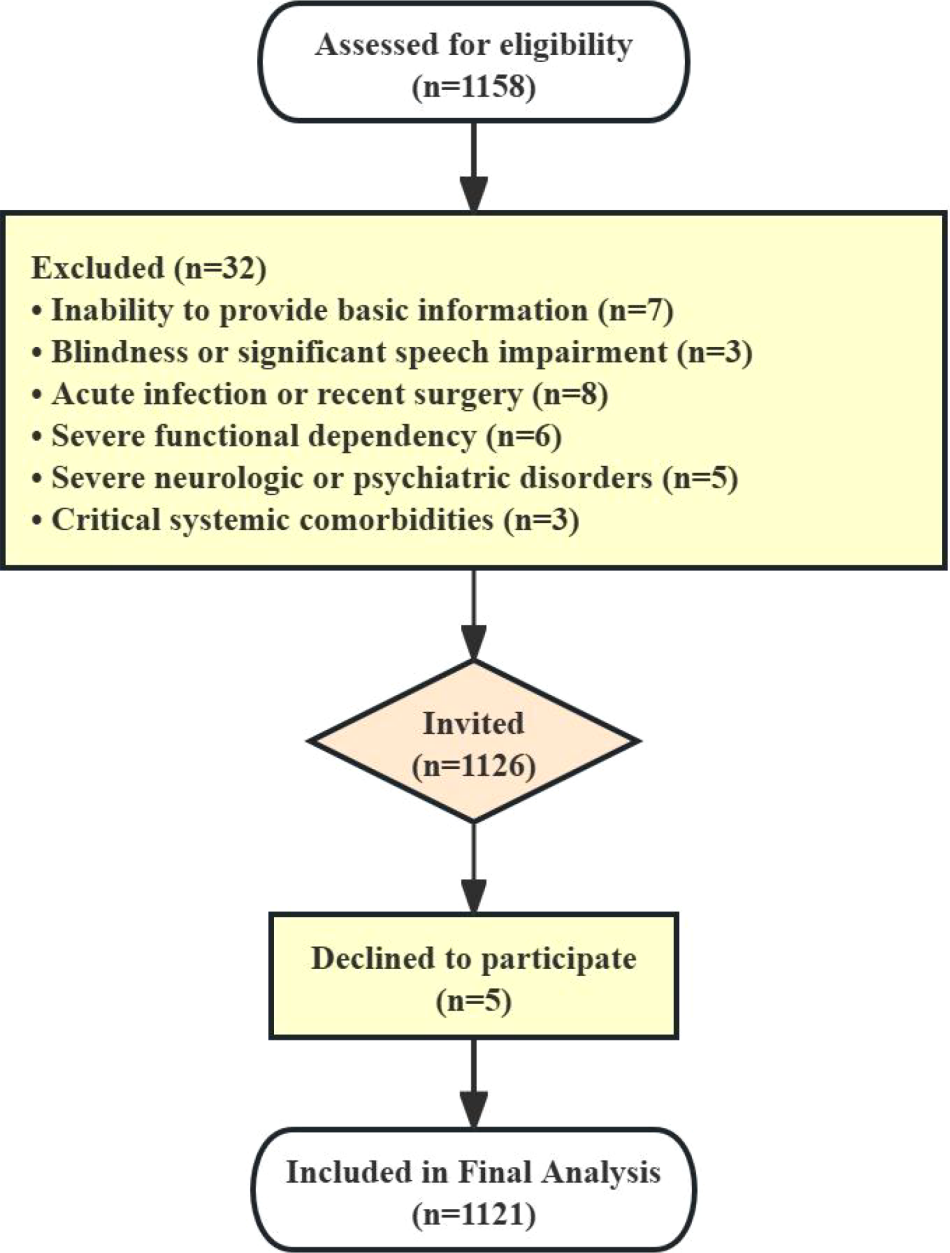
Flow diagram of participant screening, enrollment, and inclusion in the study. From August 2023 to April 2024, 1158 inpatients were consecutively screened for eligibility. Thirty-two patients were excluded based on predefined criteria. Of the 1126 eligible patients invited, 5 declined participation, resulting in a final analytic sample of 1121 participants.

Following a standardized procedure, all eligible inpatients admitted during this period were screened against the inclusion and exclusion criteria upon admission. Those inpatients who met all criteria were invited to participate. The inclusion criteria were: (1) age≥45 years; (2) absence of any acute illnesses within the past 4 weeks; (3) overall health status deemed stable by the primary physician; and (4) sufficient audiovisual capacity to complete assessments. Exclusion criteria were: (1) inability to provide basic demographic information; (2) blindness or significant speech impairment; (3) acute infection or recent surgery; (4) severe functional dependency; (5) severe neurologic or psychiatric disorders; and (6) critical systemic co-morbidities, including vital organ failure, malignancy, or an expected survival < 1 year. The participant recruitment process is summarized in a flowchart (**Figure 1**). The study was approved by the Institutional Ethics Committee of the Fifth Affiliated Hospital of Sun Yat-sen University [No. ZDWY (2024); Lunzi No. (K169-1)], and all subjects provided written informed consent.

### 2.2 Clinical data and blood tests

Participants completed a detailed questionnaire upon enrollment, providing information on basic parameters, social demographics, and medical history, including age, gender, height, weight, education level, employment status, smoking history, and disease and medication history. Height and weight were measured with participants in underwear without shoes. Body mass index (BMI) was calculated as weight in kilograms divided by the square of height in meters (kg/m²). Blood pressure was measured after a 10-minute rest, and the average value was recorded. Venous blood was collected after a 10-hour fast for biochemical analysis, including routine hematology, serum creatinine, fasting blood glucose, total serum protein, serum albumin, and lipid profiles.

### 2.3 Depression and anxiety

Depression was assessed using the observer-rated Hamilton Depression Rating Scale (HAMD), which was designed to measure depressive symptom severity (22). Most items use a 5-point scale (0–4), while some use a 3-point scale (0–2). Depression severity scores were: 7–17, suggest possible depression; 17–20 indicate mild depression; and > 20 signify moderate-to-severe depression. Higher scores indicate greater severity. Anxiety was assessed using the Hamilton Anxiety Rating Scale [HAM-A] (23). This 14-item scale uses a 5-point scale (0–4 per item; total range 0-54). Anxiety severity scores were: > 7, indicate anxiety symptoms; 7–13, indicate mild anxiety; 14–21, indicate moderate anxiety; and > 21, indicate severe anxiety.

### 2.4 Assessment of Cognitive Function

Cognitive function was evaluated using the Montreal Cognitive Assessment [MoCA] (24, 25). The Montreal Cognitive Assessment (MoCA) evaluates multiple cognitive domains, including memory, language, attention, and executive function. The maximum score is 30 points, with a score above 26 considered normal. An additional point is added for individuals with fewer than 12 years of education.

### 2.5 Sleep quality

Sleep quality was evaluated using the Pittsburgh Sleep Quality Index [PSQI] (26). This reliable and valid instrument measures the following seven dimensions of sleep: subjective quality; duration; latency; disturbances; efficiency; daytime dysfunction; and medication use. Each dimension is scored 0–3, yielding a total score from 0–21. A score of 7 or higher indicates a sleep disorder with higher scores denoting poorer sleep quality.

### 2.6 FRAIL Scale

Frailty was assessed using the FRAIL scale (27, 28). This instrument consists of the following five self-reported components: fatigue (frequent tiredness in the past 4 weeks); resistance (difficulty climbing 10 stairs unaided); walking ability (difficulty walking 500–600 meters unaided); co-morbidity (five or more diagnosed conditions, e.g., hypertension, diabetes, and stroke); and weight loss (≥ 5% body weight loss in the past year). Scores range from 0–5 with 0 indicating robust health, 1–2 indicating pre-frailty, and ≥ 3 indicating frailty.

### 2.7 Nutrition and activities of daily living

The evaluation of nutrition risk was performed with the Nutrition Risk Screening (NRS)-2002 (29). This instrument evaluates the following five parameters: (1) unexplained weight loss in the past 3 months; (2) BMI; (3) appetite; (4) disease severity; and (5) age over 70 years. A total score < 3 indicates no nutritional risk, while a score ≥ 3 signifies high nutritional risk, indicating the need for nutritional support. Activities of daily living were evaluated using the Barthel Index (29, 30). Scores range from 0–100 with lower scores indicating greater impairment in daily activities.

All assessment scales, including the HAMD, MoCA, and other instruments, were administered in person by trained research assistants. Prior to the study commencing, all raters completed a standardized training session on the proper administration and scoring of each tool to ensure assessment consistency and reliability.

### 2.8 Statistical analysis

Normality was assessed using the Kolmogorov-Smirnov test. Normally distributed data are presented as mean ± standard deviation (SD) and compared using one-way analysis of variance (ANOVA). Non-normally distributed data are presented as median (interquartile range) and compared using the Kruskal-Wallis H-test. Categorical data are presented as counts and compared using the χ^2^ test or Fisher probabilities. Correlations between variables were analyzed using Spearman’s correlation coefficient. Multiple linear regression analysis was employed to adjust for potential confounding variables. Statistical significance was defined as a *P*-value < 0.05. All analyses were performed using SPSS 26.0.

## 3 Results

### 3.1 Participant characteristics

Between August 2023 and April 2024, a total of 1158 participants were screened. After applying the inclusion and exclusion criteria, a final sample of 1121 participants was enrolled and included in the analysis, which exceeded the initial target sample size. The cohort had a mean age of 64.67 years (SD = 10.86) and was comprised of 659 (58.8%) women and 462 (41.2%) men. Most participants (57.28%) had a secondary education or below, while 22.39% completed high school and 20.33% held a university degree or above. Nearly one-half (47.3%) were unemployed or homemakers, followed by retirees (38.2%), laborers (8.5%), and technical workers or cadres (6.0%).

### 3.2 Basic data and clinical variables of participants based on HAMD and HAM-A groups

The basic and clinical characteristics according to the HAMD scores (<17, 17–20, and ≥ 20) are shown in **Table 1**. All variables, except systolic blood pressure (SBP), diastolic blood pressure (DBP), polypharmacy, fasting blood glucose (FBG), total cholesterol (TC), low-density lipoprotein (LDL), serum creatinine (SCr), total protein (TP) and C-reactive protein (CRP) were significantly different among the three HAMD groups (all *P* < 0.05). The following parameters increased in all participants with progression from low-to-high HAMD scores (all *P* < 0.05): age, serum cystatin C (CYSC), high-density lipoprotein (HDL), Fazekas score, FRAIL scale score; PSQI, and NRS-2002; while the BMI, education level, hemoglobin (HGB), uric acid (UA), estimated glomerular filtration rate (eGFR), triglycerides (TG), albumin (ALB), MoCA, and activities of daily living (ADL) decreased.

**Table 1:** Overall variables of the participants based on HAMD groups.

Variable	HAMD			
	<17(n=848)	17-20(n=165)	≥20 (n=58)	P value
Age (years)	63.21 ± 10.33	69.40 ± 10.95^**^	69.40 ± 10.45^**^	<0.001
Gender				<0.001
Male (%)	371 (43.8)	59 (35.8)	10 (17.2)	
BMI (kg/m²)	24.37 ± 3.46	23.44 ± 3.78^**^	21.89 ± 3.00^**##^	<0.001
SBP (mmHg)	126.72 ± 13.11	128.7 ± 13.29	128.17 ± 14.82	0.164
DBP (mmHg)	78.19 ± 9.31	77.26 ± 9.15	75.59 ± 8.21	0.071
Occupational status				<0.001
Unemployed/housewife/house husband	359 (43.2)	99 (61.1)	38 (66.7)	
Retirement pension	80 (9.6)	8 (4.9)	1 (1.8)	
Laborer	334 (40.2)	52 (32.1)	16 (28.1)	
Technical worker and cadre	58 (7.0)	3 (1.8)	2 (3.6)	
Education level (years)	10.01 ± 4.19	8.82 ± 4.45^**^	7.38 ± 5.05^**#^	<0.001
Cigarette smoking				0.011
Never smoker	721 (85.7)	141 (86.5)	57 (98.3)	
Former smoker	30 (3.6)	11 (6.7)	0	
Current smoker	90 (10.7)	11 (6.7)	1 (1.7)	
Illness (n, %)				0.020
None	187 (22.2)	21 (12.9)	8 (13.8)	
One or two diseases	291 (34.6)	53 (32.5)	21 (36.2)	
Three or more diseases	363 (43.2)	89 (54.6)	29 (50.0)	
Polypharmacy				0.086
Yes	140 (16.8)	39 (23.9)	12 (20.7)	
HGB (g/L)	132.35 ± 15.90	127.8 ± 15.62^**^	125.00 ± 15.90^**^	<0.001
FBG (mmol/L)	5.58 ± 1.60	5.68 ± 1.74	5.54 ± 1.28	0.763
SCr (mmol/L)	77.81 ± 22.98	78.90 ± 26.49	79.17 ± 29.09	0.812
eGFR(ml/min.1.73m²)	82.31 ± 16.85	76.74 ± 19.04^**^	74.86 ± 20.84^**^	<0.001
UA (μmol/L)	356.17 ± 99.94	325.9 ± 96.97^**^	300.27 ± 66.66^**^	<0.001
CYSC (mg/L)	0.90 ± 0.20	0.95 ± 0.21^**^	0.96 ± 0.27^*^	0.005
TG (mmol/L)	1.59 ± 1.34	1.39 ± 0.78	1.18 ± 0.45^*^	0.014
TC (mmol/L)	4.82 ± 1.15	4.83 ± 1.31	4.78 ± 1.08	0.957
HDL (mmol/L)	1.28 ± 0.36	1.35 ± 0.42^*^	1.41 ± 0.43^*^	0.007
LDL (mmol/L)	2.87 ± 0.98	2.85 ± 1.14	2.82 ± 1.01	0.934
TP (g/L)	67.49 ± 5.08	67.14 ± 5.29	67.23 ± 5.60	0.699
ALB (g/L)	40.64 ± 3.01	39.61 ± 3.76^**^	39.73 ± 3.42^*^	<0.001
CRP (mg/L)	1.04 (0.07-2.50)	1.05 (0.18-2.37)	0.55 (0.07-0.95)	0.271
Fazekas scores	1.14 ± 0.65	1.27 ± 0.71	1.47 ± 0.84^*^	0.041
Frailty scores	0.96 ± 1.02	1.84 ± 1.18^**^	2.45 ± 1.18^**##^	<0.001
MoCA	20.68 ± 5.28	16.93 ± 5.99^**^	15.24 ± 6.29^**#^	<0.001
PSQI	8.43 ± 3.86	12.98 ± 3.32^**^	14.97 ± 3.50^**##^	<0.001
ADL	94.89 ± 9.62	87.04 ± 17.23^**^	82.50 ± 17.65^**#^	<0.001
NRS	0.00 (0.00-1.00)	1.00 (0.00-2.00)^**^	1.00 (0.00-2.00)^**^	<0.001

Normally distributed data are expressed as the mean ± SD and the differences in data were examined using one-way analysis of variance (ANOVA). Data that were not normally distributed are shown as a median and interquartile range. Comparisons of these variables among groups were performed using the Kruskal–Wallis H-test. Least significant difference or Dunnett’s T3 analyses were used for comparisons between pairs of groups according to the homogeneity of variance test. *P*-values are the outcomes of ANOVA or the Kruskal–Wallis H-test. *#Significant difference (*P* < 0.05). **##Significant difference (*P* < 0.01) when compared to the < 17 and 17–20 groups. ADL, activities of daily living; ALB, serum albumin; CRP, C-reactive protein; CYSC, cystatin C; BMI, body mass index; DBP, diastolic blood pressure; eGFR, estimated glomerular filtration rate; FBG, fasting blood glucose; HDL, high-density lipoprotein; HGB, hemoglobin; LDL, low-density lipoprotein; NRS, nutritional risk; SBP, systolic blood pressure; SCr, serum creatinine; TC, total cholesterol; TG, triglycerides; TP, total serum protein; UA, uric acid.

The variables of the participants are shown according to HAM-A groups in **Table 2**. A higher HAM-A score was significantly associated with higher age, HDL, FRAIL score, PSQI and NRS-2002 but a lower DBP, education level, HGB, eGFR, UA, TG, ALB, MoCA, and ADL. There were no significant differences in BMI, SBP, FBG, SCr, CYSC, TC, LDL, TP, or Fazekas score with respect to HAM-A scores.

**Table 2:** Overall variables of the participants based on HAM-A groups.

Variable	HAMA			
	<14(n=254)	14-21(n=662)	≥21(n=155)	P value
Age (years)	61.06 ± 9.56	64.74 ± 10.56^**^	69.06 ± 11.38^**##^	<0.001
Gender				<0.001
Male (%)	139 (54.7)	267 (40.3)	34 (21.9)	
BMI ((kg/m²)	24.19 ± 3.05	24.19 ± 3.62	23.55 ± 3.92	0.119
SBP (mmHg)	127.08 ± 13.76	127.05 ± 13.11	127.42 ± 13.07	0.951
DBP (mmHg)	79.39 ± 9.45	77.77 ± 9.37^*^	76.08 ± 7.89^**#^	0.002
Occupational status				<0.001
Unemployed/housewife/house husband	74 (30.0)	331 (51.0)	91 (59.1)	
Retirement pension	37 (15.0)	44 (6.8)	8 (5.2)	
Laborer	106 (42.9)	246 (37.9)	50 (32.5)	
Technical worker and cadre	30 (12.1)	28 (4.3)	5 (3.2)	
Education level	11.19 ± 3.65	9.51 ± 4.31^**^	7.95 ± 4.64^**##^	<0.001
Cigarette smoking				0.029
Never smoker	212 (84.1)	562 (85.7)	145 (94.2)	
Former smoker	9 (3.6)	30 (4.6)	2 (1.3)	
Current smoker	31 (12.3)	64 (9.8)	7 (4.5)	
Illness				0.012
None	70 (27.8)	124 (18.9)	22 (14.3)	
One or two diseases	79 (31.3)	229 (34.9)	57 (37.0)	
Three or more diseases	103 (40.9)	303 (46.2)	75 (48.7)	
Polypharmacy				0.010
Yes	32 (12.9)	121 (18.6)	38 (24.8)	
HGB (g/L)	135.91 ± 14.07	130.5 ± 16.33^**^	126.52 ± 15.76^**##^	<0.001
FBG (mmol/L)	5.47 ± 1.45	5.62 ± 1.65	5.66 ± 1.64	0.388
SCr (mmol/L)	79.10 ± 20.95	77.67 ± 24.47	77.99 ± 25.93	0.723
eGFR(ml/min.1.73m²)	83.80 ± 15.76	81.28 ± 17.54	75.60 ± 19.50^**##^	<0.001
UA (μmol/L)	364.79 ± 91.98	349.31 ± 103.12^*^	318.23 ± 86.11^**##^	<0.001
CYSC (mg/L)	0.88 ± 0.15	0.91 ± 0.22	0.93 ± 0.20	0.054
TG (mmol/L)	1.67 ± 1.58	1.53 ± 1.18	1.33 ± 0.78^*^	0.037
TC (mmol/L)	4.89 ± 1.04	4.81 ± 1.19	4.73 ± 1.27	0.370
HDL (mmol/L)	1.25 ± 0.35	1.29 ± 0.37	1.38 ± 0.42^**#^	0.004
LDL (mmol/L)	2.93 ± 0.86	2.86 ± 1.02	2.74 ± 1.15	0.189
TP (g/L)	67.43 ± 5.01	67.37 ± 5.14	67.60 ± 5.36	0.885
ALB (g/L)	40.85 ± 2.99	40.37 ± 3.22^*^	40.01 ± 3.30^*^	0.026
CRP (mg/L)	0.99 (0.07-2.13)	1.02 (0.07-2.74)	0.90 (0.25-1.92)	0.102
Fazekas scores	1.05 ± 0.63	1.21 ± 0.66	1.23 ± 0.74	0.058
Frailty scores	0.61 ± 0.79	1.18 ± 1.09^**^	1.94 ± 1.27^**##^	<0.001
MoCA	22.19 ± 4.51	19.58 ± 5.66^**^	16.90 ± 6.18^**##^	<0.001
PSQI	6.53 ± 3.55	9.64 ± 3.83^**^	13.65 ± 3.61^**##^	<0.001
ADL	97.15 ± 5.62	93.16 ± 11.90^**^	85.61 ± 17.17^**##^	<0.001
NRS	0.00 (0.00-1.00)	0.00(0.00-1.00)^**^	1.00 (0.00-2.00)^**##^	<0.001

Abbreviations as in **Table 1**. Normally distributed data are expressed as the mean ± SD and the differences in these data were examined using one-way analysis of variance (ANOVA). Data that were not normally distributed are shown as a median and interquartile range. Comparisons of these variables among groups were performed using the Kruskal–Wallis H-test. Least significant difference or Dunnett’s T3 analyses were used for comparisons between pairs of groups according to the homogeneity of variance test. *P*-values are the outcome of ANOVA or the Kruskal–Wallis H-test. *#Significant difference (*P* < 0.05). **##Significant difference (*P* < 0.01) when compared to <14 and 14–21 groups.

**Figure 2:**
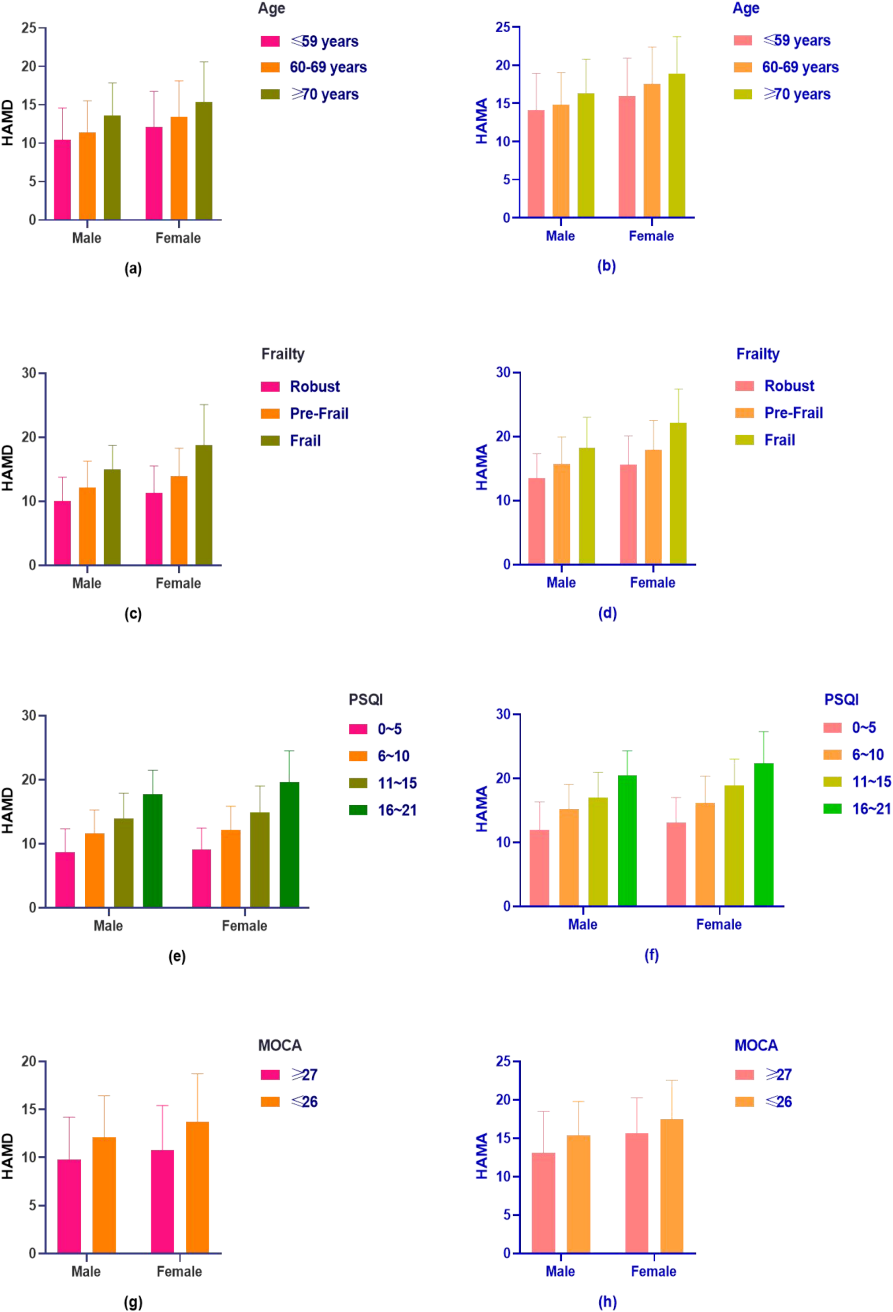
Distribution of HAMD and HAM-A scores across key subgroups. The figure illustrates the distribution patterns of depression (HAMD; left panels) and anxiety (HAM-A; right panels) scores among participants, stratified by **(a, b)** age, **(c, d)** frailty, **(e, f)** sleep quality (PSQI), and **(g, h)** cognitive function (MoCA). Each panel compares the score distributions between men and women within the specified categories.

As shown in **Figure 2**, HAMD and HAM-A scores varied significantly across different patient subgroups. Subsequent *post-hoc* tests confirmed that all inter-group comparisons were statistically significant (all *P* < 0.001). This finding included the following comparisons: older and middle-aged patients (**Figures 1a, b**); robust, pre-frail, and frail groups (with each group differing from the others; **Figures 1c, d**); good and poor sleep quality groups (**Figures 1e, f**); and normal cognition and mild cognitive impairment groups (**Figures 1g, h**).

### 3.3 Correlation of HAMD and HAM-A scores with basic characteristics and clinical variables

Correlations between HAMD and HAM-A scores and basic and clinical parameters are shown in **Table 3**. There was a weak correlation between the HAMD scores and age, occupational status, education level, HGB, MoCA, ADL, and NRS-2002 (0.2 < r < 0.4; *P* < 0.01). There was a moderate correlation between the HAMD scores and FRAIL score (r = 0.401; *P* < 0.01). There was a strong correlation between the HAMD score and PSQI (r = 0.605; *P* < 0.01). The HAMA score was correlated with age, gender, education level, HGB, MoCA, ADL, NRS-2002, FRAIL score (0.2 < r < 0.4; *P* < 0.01), and PSQI (r = 0.553; *P* < 0.01).

**Table 3:** Correlation of HAMD and HAM-A scores with clinical variables, Rp (Spearman’s correlation coefficient).

Variable	HAMD		HAMA	
	r_p_	p	r_p_	p
Age (years)	0.286^**^	<0.001	0.237^**^	<0.001
Gender	0.143^**^	<0.001	0.218^**^	<0.001
BMI ((kg/m²)	-0.168^**^	<0.001	-0.054	0.080
SBP (mmHg)	0.011	0.712	-0.007	0.827
DBP (mmHg)	-0.129^**^	<0.001	-0.110^**^	<0.001
Occupational status	-0.251^**^	<0.001	-0.171^**^	<0.001
Education level	-0.229^**^	<0.001	-0.216^**^	<0.001
Cigarette smoking	-0.067^*^	0.029	-0.076^*^	0.013
Illness	0.131^**^	<0.001	0.110^**^	<0.001
Polypharmacy	0.096^**^	0.002	0.103^**^	0.001
HGB (g/L)	-0.223^**^	<0.001	-0.204^**^	<0.001
FBG (mmol/L)	0.034	0.269	0.050	0.103
SCr (mmol/L)	-0.023	0.462	-0.081^**^	0.009
eGFR (ml/min. 1.73m²)	-0.158^**^	<0.001	-0.117^**^	<0.001
UA (μmol/L)	-0.199^**^	<0.001	-0.160^**^	<0.001
CYSC (mg/L)	0.128^**^	<0.001	0.052	0.114
TG (mmol/L)	-0.104^**^	0.001	-0.056	0.068
TC (mmol/L)	-0.056	0.068	-0.055	0.072
HDL (mmol/L)	0.076^*^	0.014	0.091^**^	0.003
LDL (mmol/L)	-0.062^*^	0.045	-0.069^*^	0.025
TP (g/L)	0.015	0.620	0.021	0.504
ALB (g/L)	-0.149^**^	<0.001	-0.098^**^	0.001
CRP (mg/L)	0.013	0.806	0.084	0.100
Fazekas scores	0.138**	0.002	0.087*	0.048
Frailty scores	0.401^**^	<0.001	0.381^**^	<0.001
MoCA	-0.366^**^	<0.001	-0.279^**^	<0.001
PSQI	0.605^**^	<0.001	0.553^**^	<0.001
ADL	-0.310^**^	<0.001	-0.282^**^	<0.001
NRS	0.311^**^	<0.001	0.214^**^	<0.001

Abbreviations as in **Table 1**. *P*-values are from analysis of variance.

*Significant difference (*P* < 0.05).

**Significant difference (*P* < 0.01).

### 3.4 Relationship between the HAMD and HAM-A scores and clinical variables using multiple linear regression analysis

Multiple linear regression (stepwise entry) identified factors independently associated with HAMD and HAM-A scores (**Table 4**). The model included parameters correlated in pairwise analysis and traditional risk factors. After controlling for confounding factors, the HAMD score had a significant and independent correlation with gender (β = 0.078, *P* = 0.017), BMI (β = -0.096, *P* = 0.003), MoCA (β = -0.141, *P* < 0.001), PSQI (β = 0.494, *P* < 0.001), NRS-2002 (β = 0.099, *P* = 0.004), and FRAIL score (β = 0.200, *P* < 0.001). The HAMA score was significantly correlated with gender (β = 0.155, *P* < 0.001), illness (β = -0.098, P = 0.006), MoCA (β = -0.114, P = 0.001), PSQI (β = 0.435, *P* < 0.001), and FRAIL score (β = 0.276, *P* < 0.001).

**Table 4:** Relationship between HAMD and HAM-A scores and clinical variables using a stepwise multiple regression model.

Variable	HAMD				HAM-A			
	Model1		Model2		Model1		Model2	
	Beta	p	Beta	p	Beta	p	Beta	p
Gender	0.080^**^	0.005	0.078^*^	0.017	0.141^**^	<0.001	0.155^**^	<0.001
BMI ((kg/m²)	-0.112^**^	<0.001	-0.096^**^	0.003	0.002	0.938	0.013	0.709
Illness	-0.017	0.571	-0.021	0.536	-0.102^**^	0.002	-0.098^**^	0.006
MoCA	-0.161^**^	<0.001	-0.141^**^	<0.001	-0.129^**^	<0.001	-0.114^**^	0.001
Frailty scores	0.231^**^	<0.001	0.200^**^	<0.001	0.285^**^	<0.001	0.276^**^	<0.001
PSQI	0.482^**^	<0.001	0.494^**^	<0.001	0.428^**^	<0.001	0.435^**^	<0.001
NRS	0.063^*^	0.046	0.099^**^	0.004	-0.023	0.500	-0.027	0.461

Beta, standardized coefficients. Model 1, unadjusted model; Model 2, fully adjusted for age, gender, BMI, SBP, DBP, HGB, FBG, TG, TC, LDL, SCr, eGFR, UC, CYSC, TP, ALB, working status, education level, illnesses, ADL, frailty scores, NRS, PSQI, and MoCA. Standardized coefficients and *P* values were the outcome of regression analyses.

*Significant difference (*P* < 0.05).

**Significant difference (*P* < 0.01).

## 4 Discussion

This study aimed to address a significant clinical gap by systematically investigating modifiable risk factors for anxiety and depression among general hospital patients. It contributes comprehensive evidence on sociodemographic, lifestyle, clinical, and biological determinants. This evidence base can inform the development of targeted interventions and support future strategies aimed at improving mental health outcomes in this patient population.

Our results confirm previous evidence linking cognitive impairment to anxiety and depression (12). After controlling for confounders, the MoCA score was significantly and independently correlated with the HAMD and HAM-A scores (all *P* < 0.01). Therefore, the results confirmed that cognitive impairment is an independent risk factor for mental disorders. Cognitive and mental disorders have been shown to have a bidirectional causal relationship (12). Mental disorders may be precursors to cognitive impairment and early non-cognitive manifestations of dementia (31, 32). Neurobiological research demonstrated that psychological disorders and dementia often have the same neuropathologic changes, particularly in the limbic system. Therefore, neurodegenerative changes within this system may result in emotion-related symptoms (33). Mechanisms potentially linking cognitive impairment and mental disorders include hypercortisolemia (34), chronic inflammation (35), and common shared risk factors, such as vascular disease, chronic illnesses, sleep disturbances, and unhealthy lifestyles (35). Our analysis, incorporating key confounders like vascular risk factors, co-morbidities, and sleep quality, strengthens previous findings by demonstrating the independent association of cognitive function with depression and anxiety.

Significantly higher PSQI scores (poorer sleep) were observed in groups with higher HAMD/HAM-A scores, which are consistent with prior research. The PSQI score remained significantly correlated with the HAMD and HAM-A scores after full adjustment. The bidirectional link between affective disorders and sleep disturbances is well-established. Nearly all patients with mental disorders have sleep disturbances. Patients with affective disorders accompanied by sleep abnormalities typically show more severe symptoms. Mechanisms linking poor sleep and mental disorders include autonomic nervous system dysregulation and abnormalities in brain regions among individuals with sleep disturbances involved in regulating emotions (1). Circadian rhythm disruption and chronic insomnia can negatively impact mood and increase the risk of anxiety and depression (10). Our study confirms that poor sleep quality is independently associated with affective disorders. Furthermore, the study results suggested that cognitive function and sleep influence emotions through both shared and distinct pathways. Thus, valuing cognitive function and improving sleep may be associated with a lower risk of affective disorders.

A strong relationship was previously confirmed between affective disorders and frailty, which agrees with the findings in the current study. Frailty scores differed significantly between HAMD/HAM-A groups (*P* < 0.01). The patients with affective disorders were more likely to have frailty, even after accounting for risk factors, such as age, gender, BMI, cigarette smoking, cognition, ADL, nutrition, and illnesses. This strong correlation was sustained after controlling for the main potential confounding factors. The mechanism underlying frailty and emotion disorders include accelerated biological aging, chronic inflammation, and shared risk factors, such as chronic illnesses, cognitive deficits, and malnutrition (14). Frail individuals are more likely to increase emotional disturbance due to functional impairment and social withdrawal (11). Given the above findings, the current study provides theoretical support for the view that effective management of frailty may be correlated with a slower progression of emotional health disturbances.

A higher BMI was associated with a lower likelihood of depression in the current study. The findings were in contrast to previous studies involving European populations in which obesity and overweight are correlated with a higher probability of depression (18, 36). The correlation between adiposity and depression in Europeans was explained by social factors, worsening physical health, and the potential appearance later in life (18). Our results align with evidence of a negative association between BMI and depression in East Asian populations (21), possibly reflecting cultural perspectives where higher body weight is sometimes perceived positively (“happy mind and fat body”). Differences in biological factors and cultural perspectives on obesity may explain the different effects observed herein. Nutritional risk (NRS-2002) was significantly associated with depression, which is consistent with the crucial role of nutrition in brain function and metabolic pathways affecting mental health (37). Nutrients, like vitamin B12, folate, iron, and essential fatty acids, are vital for neurotransmission, enzymatic activity, and cellular processes (38). The significant association between co-morbidities and anxiety aligns with prior research (39). Anxiety can heighten awareness of physical symptoms, reduce treatment adherence, and increase complications in chronic illness. Conversely, the symptom burden and functional limitations caused by illness can worsen anxiety.

Females, especially elderly females, exhibited a higher risk of affective disorders than males, which is consistent with numerous studies (40–42). Proposed explanations for the increased risk of affective disorders in females include gender differences in response to inflammation and stress, sexual differentiation of brain structures and the neuroimmune system, hormonal differences, social and cultural norms, different gender roles, and disadvantages and empowerment throughout life. However, the precise mechanisms underlying the gender differences in affective disorder risk require further elucidation. A systematic understanding of the impact of gender on affective disorders could benefit prevention and treatment strategies, warranting larger confirmatory studies.

This study explored modifiable risk factors for anxiety and depression in inpatients by integrating sociodemographic, lifestyle, clinical, and biological data. The resulting insights provide practical tools for bedside risk stratification and identify potential intervention targets, thereby aiding in clinical management. Nevertheless, several limitations need to be emphasized. First, due to the cross-sectional design of the study, the direction of the causal relationship could not be established from the present data set. The use of longitudinal or experimental designs in future research is critical for inferring causality. Second, regarding generalizability, all participants were recruited from one hospital. Although the sample reflects a typical inpatient profile, the findings may not be fully applicable to outpatients, community populations, or healthcare settings with markedly different demographics or resources. Future multi-center studies across diverse regions in China are warranted to enhance external validity. Finally, owing to limitations in measurement methods and variable selection, this study did not analyze some potentially influential factors, such as specific in-hospital treatments (e.g., surgical procedures). The impact on psychological outcomes could not be assessed and should be incorporated into future analyses.

## 5 Conclusion

This study analyzed the correlation between depression and anxiety with multiple risk factors, including sociodemographic, lifestyle, and clinical and biological domains in hospitalized patients. The analysis revealed specific correlation patterns. For example, a higher PSQI score (β = 0.435, *P* < 0.001) and FRAIL score (β = 0.276, *P* < 0.001) were positively correlated with greater anxiety severity. Other significant factors independently associated with increased symptom burden included lower BMI, multimorbidity, poorer cognitive function, and nutritional risk (all *P* < 0.05). These quantified associations underscore the necessity of early screening and preventive strategies focused on modifiable factors. Clinical support should be prioritized for higher-risk subgroups, such as women and older adults, who present with compounded vulnerabilities, including affective disorders, fatigue, and sleep disturbances. Interventions aimed at improving sleep, mitigating frailty and illness progression, preserving cognitive function, and promoting healthy lifestyles hold promise for an association with a reduced burden of mental disorders. Further research is needed to confirm and expand upon these findings.

## Data Availability

The original contributions presented in the study are included in the article/supplementary material. Further inquiries can be directed to the corresponding author.
